# Retraction Note: A novel microRNA identified in hepatocellular carcinomas is responsive to LEF1 and facilitates proliferation and epithelial-mesenchymal transition via targeting of NFIX

**DOI:** 10.1038/s41389-021-00336-9

**Published:** 2021-07-15

**Authors:** Yaqi Hu, Xu Guo, Jinxia Wang, Yankun Liu, Huijie Gao, Hongxia Fan, Xiangyang Nong, Xi Yang, Min Liu, Shengping Li, Hua Tang

**Affiliations:** 1 Tianjin Life Science Research Center and Department of Pathogen Biology, Collaborative Innovation Center of Tianjin for Medical Epigenetics, School of Basic Medical Sciences, Tianjin Medical University, 300070 Tianjin, China; 2 The Cancer Institute, Tangshan People’s Hospital, 063001 Tangshan, China; 3 Department of Hepatobiliary Oncology, State Key Laboratory of Oncology in Southern China, Cancer Center, Sun Yat-sen University, 510060 Guangzhou, China

Retraction to: *Oncogenesis* 10.1038/s41389-017-0010-x, published online 23 February 2018

The Editor-in-Chief has retracted this article because of significant concerns regarding a number of Figures presented in this work: Figures 3, 5 and 6 appear to contain multiple duplicated panels. The Editor-in-Chief therefore no longer has confidence in the integrity of the data in this article.

Yaqi Hu, Xu Guo, Jinxia Wang, Yankun Liu, Huijie Gao, Hongxia Fan, Xi Yang, Shengping Li and Hua Tang have agreed to this retraction; Xiangyang Nong and Min Liu have not responded to any correspondence from the Publisher about this retraction.

